# Magnitude and associated factors of dyslipidemia among patients with severe mental illness in dire Dawa, Ethiopia: neglected public health concern

**DOI:** 10.1186/s12872-023-03327-3

**Published:** 2023-06-13

**Authors:** Dilnessa Fentie, Shegaye Yibabie

**Affiliations:** Medical department, medical and health sciences college, Dire Dawa University, Dire Dawa, Ethiopia

**Keywords:** Dyslipidemia, Dire Dawa, Schizophrenia, Hypercholesterolemia, Atherosclerosis

## Abstract

**Background:**

Lipid metabolism abnormalities are an emerging risk factor for cardiovascular diseases. Due to the nature of the condition and their unhealthy lifestyles, patients with mental illnesses have a doubled risk of morbidity and mortality from dyslipidemia compared to the general population. To our knowledge the magnitude of dyslipidemia in patients with mental illnesses in the eastern Ethiopia has not been reported in the literature to date. Therefore, the aim of the study was to assess and compare the magnitude of dyslipidemia and its predictors among patients with severe mental illnesses and non-mentally ill control patients.

**Methods:**

Nighty six subjects with serious psychiatric disorders and nighty six matched non-psychiatric control subjects who had no history of psychiatric illness were underwent a lipid profile test in Dire Dawa referral hospital, Ethiopia. The mentally ill clients were 18 years of age and older with schizophrenia, major depression, and bipolar disorders. Exposed study subjects were matched to control by age and sex. The data were cleaned and analyzed using SPSS software. A binary logistic regression model was used to determine the factors related to the magnitude of dyslipidemia. Both the crude odds ratio and the adjusted odds ratio with a 95% confidence interval were estimated.

**Results:**

The magnitude of dyslipidemia among mentally ill patients was significantly higher (63.54%) compared to non-exposed controls (31.9%) in the subjects studied. In multiple logistic regression, urban dwellers were six times (AOR = 6.14, 95% CI: 1.2, 16) more likely at risk of developing dyslipidemia compared to rural participants. Similarly, physically inactive participants were nearly two-times (AOR = 1.8, 95% CI: 1.1, 12.9) more likely to develop dyslipidemia compared to physically active study participants. Moreover, study participants who had raised body mass index were 2.1 times (AOR = 2.1, 95% CI: 1.17, 15.3) more likely having dyslipidemia than their counterparts.

**Conclusions:**

This study revealed that the prevalence of dyslipidemia is higher among mentally ill patients compared to non-mentally ill control study participants. Place of residence, physical inactivity, and raised BMI were significantly associated with dyslipidemia. Therefore, intensive screening of patients for dyslipidemia and its components is necessary during follow-up.

## Introduction

A condition of lipid metabolism abnormality is dyslipidemia, characterized by raised levels of total cholesterol (TC), high-density lipoprotein cholesterol (LDL-C), low-density lipoprotein cholesterol (HDL-C), and elevated triglycerides, alone or in combination (TG [1]. The body’s level of cholesterol is controlled by both LDL-C and HDL-C. Stroke and myocardial infarction risk can both be raised by an imbalance between the two lipid components. Due to the accumulation of plaque in the arteries, high LDL-C is linked to an increased risks of developing atherosclerotic CVD. The person who has high levels of HDL-C, however, has an atherosclerosis defense mechanism [2].

Serious mental illnesses such as schizophrenia, bipolar disorder, and major depression accompany an excess burden of cardiovascular morbidity and mortality [3]. There is some debate and complexity surrounding the connection between dyslipidemia and mental illnesses. According to studies, people with prevalent mental disorders are more likely to have dyslipidemia [4]. Potential risk factors for dyslipidemia in mental patients include improper dietary habits, excessive alcohol use, poor sleep hygiene, physical inactivity, psychotropic medicines, and smoking, all of which are more prevalent in certain psychiatric patients [5, 6].

The majority of mental health conditions, such as schizophrenia, bipolar disorder, and major depression disorder, are associated with an extra burden of cardio-metabolic morbidity and mortality [7]. Antipsychotics, mood stabilizers, and several antidepressants are among the many regularly used psychiatric drugs that have been independently linked to cardio-metabolic risk factors such as insulin resistance, obesity, and dyslipidemia [8]. The antipsychotic medications clozapine and olanzapine have been linked in case studies to dyslipidemia that resolves when the medication is withdrawn [9]. These findings imply that patients with schizophrenia or mood disorders taking the most regularly prescribed antipsychotic drugs are more likely to acquire dyslipidemia [10, 11].

Due to increased consumption of bad diets, decreased physical activity, increased substance use, urbanization, and obesity, the burden of dyslipidemia in patients with mental disorders is continuously rising on a global scale [6, 7]. According to a multicenter study from China, the frequency of dyslipidemia among patients with mental illnesses rose with time, rising from 4.88% to 2005 to 19.66% in 2018. When it comes to patients with schizophrenia, recurrent depressive disorder, and bipolar disorder, the prevalence of dyslipidemia was 18.36%, 14.70%, and 11.63%, respectively [12]. Furthermore, according to research from the Middle East, dyslipidemia is quite common, with incidence rates ranging from 38.6% in Lebanon to 78.6% in Afghanistan. Similarly, the prevalence of dyslipidemia varied from 15% in Saudi Arabia, Morocco, and Afghanistan to 69% in those same countries [13, 14].

In Sub-Saharan Africa, the prevalence of dyslipidemia was 25.5%. Higher levels of LDL cholesterol (37.3%), higher levels of total cholesterol (28.5%), and lower plasma HDL cholesterol (17.0%) were found [15]. Additionally, a single study from southern Ethiopia found that 58.4% of mentally ill patients had dyslipidemia [16]. In addition, the prevalence of dyslipidemia was reported in non-mentally ill study participants in different parts of the Ethiopia region; 68.1% in southwest Ethiopia [17], 66.7% in northern Ethiopia [18], and 59% in central Ethiopia [19].

Worldwide, dyslipidemia is still substantially underdiagnosed and undertreated, and many people, particularly in developing nations like Ethiopia, lack access to lipid-lowering medications [20, 21].

Designing effective strategies to address the impact of dyslipidemia on cardiovascular disease depends critically on an understanding of the magnitude of dyslipidemia and its possible implications for seriously sick mental ill patients. According to our knowledge, there is not much data on the prevalence and contributing causes of dyslipidemia in severe mental ill patients. We look into the claim that sick and mentally ill patients have a higher magnitude of dyslipidemia than people who are not suffering from mental illnesses.

The aim of the study was to estimate and compare the magnitude of dyslipidemia, and factors associated with dyslipidemia among patient with severe mentally illnesses and non-mentally ill control participants.

## Methods

### Study setting and design

Dire Dawa, one of the major cities in Ethiopia, has one referral hospital (DCRH), established in 1956 E.C. Service is provided through the inpatient and outpatient departments of the mental clinic. At the moment, there are around 700 psychiatric patients receiving antipsychotic medications and going to follow-up appointments monthly at the outpatient department. More than 4,500 individuals receive services from the clinic annually. A comparative cross-sectional study was employed among patients with severe mental illnesses and non-exposed individuals to assess dyslipidemia and its associated factors in patients attending from January 5 to June 10, 2021.

### Source and study population

#### Severe mental illness

Clients with the diagnosis of Schizophrenia, schizoaffective disorder, major depressive disorder, and bipolar disorder.

Non-exposed individuals (non-mentally ill controls): age-and sex-matched individuals, did not have any mental disorder diagnoses, and who attended outpatient departments.

### Eligibility of the study

Both groups of the study participants who were 18 years or older were eligible for this study. Those who used hormonal contraceptives, had a history of pregnancy, heart failure, or unstable mental health conditions were not included in the study.

### Study participants and Sampling procedures

The double population proportion formula was used to get the study participants. Based on previous studies [22, 23], we calculated that the prevalence of cardiometabolic risk was 28.9% in the mentally ill exposed group and 12.5% in the non-exposed group to make the sentence more interesting. The estimated sample size at 80% power, 95% confidence, and a 10% non-response rate, required sample size was 192 study participants (96 mentally ill patients and 96 non-exposed controls). A consecutive sampling procedure was used to select the study participants.

### Data collection instrument and procedures

Through interviews with trained nurses and the use of a structured questionnaire, information on socio-demographic, behavioral, and clinical aspects was gathered. The questionnaire was adapted from related literature.

### Physical and blood pressure measures

Body mass index (BMI) was computed using weight in kilograms divided by the square of height in meters. After the participant had been seated for 5 min, blood pressure was taken using a calibrated manual sphygmomanometer. According to ATS III standards, hypertension was defined as systolic blood pressure (SBP ≥ 130 mmHg) or diastolic blood pressure (DBP ≥ 85 mmHg).

### Biochemical analysis and procedure

A trained technician followed stringent standard operating procedures and collect 5 milliliters of venous blood specimen from each study participant after they had fasted for the previous night. The sample was then stored at room temperature for 30 min before being centrifuged using a Rotanta 960 centrifuge for 5 min at a speed of 4000 revolutions per minute. By using the direct end- point enzymatic approach, the ABX Pentra 400 automated clinical chemistry analyzer (Spain) examined the serum lipid profiles (TC, HDL-c, LDL-c, and TGs).

### Data quality assurance

To ensure uniformity, the questions were translated into local languages. The investigators reviewed each questionnaire daily to ensure its accuracy, completeness, and clarity. The pretest was conducted prior to the actual data collection time. Normal operating procedures were followed for all laboratory tests (SOPs). Commercially manufactured quality control (QC) samples were used to test the accuracy of laboratory equipment’s.

### Term definitions

According to National Cholesterol Education Program Adult Treatment Panel III (NCEPATP III) standards, dyslipidemia is the presence of at least one or more lipid profile abnormalities from the following ranges: high TC at least 200 mg/dl, high LD at least 100 mg/dl, high TG at least 150 mg/dl, or low HDL at most 40 mg/dl [24, 25].

### Statistical analysis

Using Epi-data software, the data were verified for accuracy before being exported to the Statistic Package for Social Science (SPSS) for analysis. An independent sample t-test was used to assess the significance mean differences in continuous measurements. Moreover, the significance differences of proportions between the two study groups were compared using Pearson’s chi-squared test. After analysis, the data were presented using texts, graphs, and tables. A binary logistic regression model was used to determine the factors related to the magnitude of dyslipidemia. Variables with a p-value of < 0.25 in the bi-variable analysis were entered into the multivariable analysis. Both the crude odds ratio and the adjusted odds ratio with a 95% confidence interval were estimated to show the strength of the associations. P-value < 0.05 in the multi-variable logistic regression analysis, association was considered significant.

## Results

### Study population characteristics

The overall number of participants in this study was 192, including 96 participants with severe mental illnesses and 96 non-mentally ill study participants. The two study groups did not significantly differ in terms of age, gender, educational attainment, alcohol use, past smoking, or levels of physical activity. More than half of (61.5%) mentally ill study participants were from rural residence areas. The remaining general characteristics of the study participants were included in the table below (Table 1).

**Table 1:** Socio-demographic characteristics of the study groups by mental illness status, Dire Dawa, 2021

Variables	Category	Cases (n = 96)	Controls(n = 96)	p- value
Sex	Male	55 (57.3%)	56(58.3%)	0.561*
	Female	41(42.7%)	40(41.7%)	
Age (mean ± SD)	-	37.18 ± 12.59	36.59 ± 13.56	0.754**
Educational status	Illiterate	45(46.9%)	26(27%)	0.076*
	Educated	51(53%)	70(72.9%)	
Marital status	Single/unmarried	35(36.5%)	33(34.4%)	0.743*
	Married	12(12.5%)	33(34.4%)	
	Divorced/separated/died	12(12.5%)	13(13.5%)	
Residence	Rural	59 (61.5%)	41(42.7%)	0.009*
	Urban	37(38.5%)	55 (57.3%)	
Employment	Unemployed	72(75.0%)	36(37.5%)	0.061*
	Employed	24(25.0%)	60(62.5%)	
Smoking	No	79 (82.3%)	82 (85.4%)	0.86*
	Yes	17 (17.7%)		
Alcohol intake	No	70(72.9%)	75 (78.1%)	0.401*
	Yes	26(27.1%)	21 (21.9%)	
Physical activity				0.521*
	Yes	39(40.6%)	29(30.2%)	
	No	57(59.4%)	67(69.8%)	
Family history of hypertension	Yes	36(37.5%)	13(13.5%)	0.082*
	No	69(71.9%)	83(86.5%)	
Family history of diabetes	Yes	27(28.1%)	13(13.5%)	0.013*
	No	68(70.8%)	83(86.5%)	
BMI(kg/m2)	< 25	42(43.8%)	20(20.8%)	0.001*
	≥ 25	54(56.3%)	76(79.2%)	

BMI: body mass index, SD: standardization, **Independent sample T test, *Pearson’s chi-square test

### Magnitude of dyslipidemia

In general, 63.54% of mentally ill study participants had dyslipidemia, compared to 31.9% of non-exposed controls (p = 0.032). Furthermore, in terms of the component of dyslipidemia, patients with severe mental illnesses were more likely to have hypercholesterolemia, higher low density lipoprotein cholesterol, and reduced HDL-C levels compared to the non-mentally ill control clients. However, serum triglyceride level were statically negligible difference between exposed and non- patients (p = 0.09) (Table 2).

**Table 2:** Prevalence and pattern of dyslipidemia by mental disorder status, Dire Dawa, 2021

Lipid Profile	Categories	Mentally ill individuals	Non-mentally ill controls	P value
Over all dyslipidemia		63.5%(95%CI:53.7–69.6)	31.9%(23.8–39.4)	0.032
TC	< 200 mg/dl ≥ 200 mg/dl	55(57.3%) 41(42.7%)	75(78.1%) 21(21.9%)	0.002
LDL-c	< 130 mg/dl ≥ 130 mg/dl	60(62.5%) 36(37.5%)	74(77.1%) 22(22.9%)	0.028
TG	< 150 mg/dl ≥ 150 mg/dl	59(61.5%) 37(38.5%)	70(72.9%) 26(27.1%)	0.09
HDL-c	≤ 40 mg/dl > 40 mg/dl	34(35.4%) 62(64.6%)	20(20.8%) 76(79.2%)	0.025

TC: Total Cholesterol; HDL-C: High Density Lipoprotein Cholesterol; LDL-C: Low Density Lipoprotein-Cholesterol; TG: Triglyceride

### Dyslipidemia patterns in various mental illnesses

**Fig.1:**
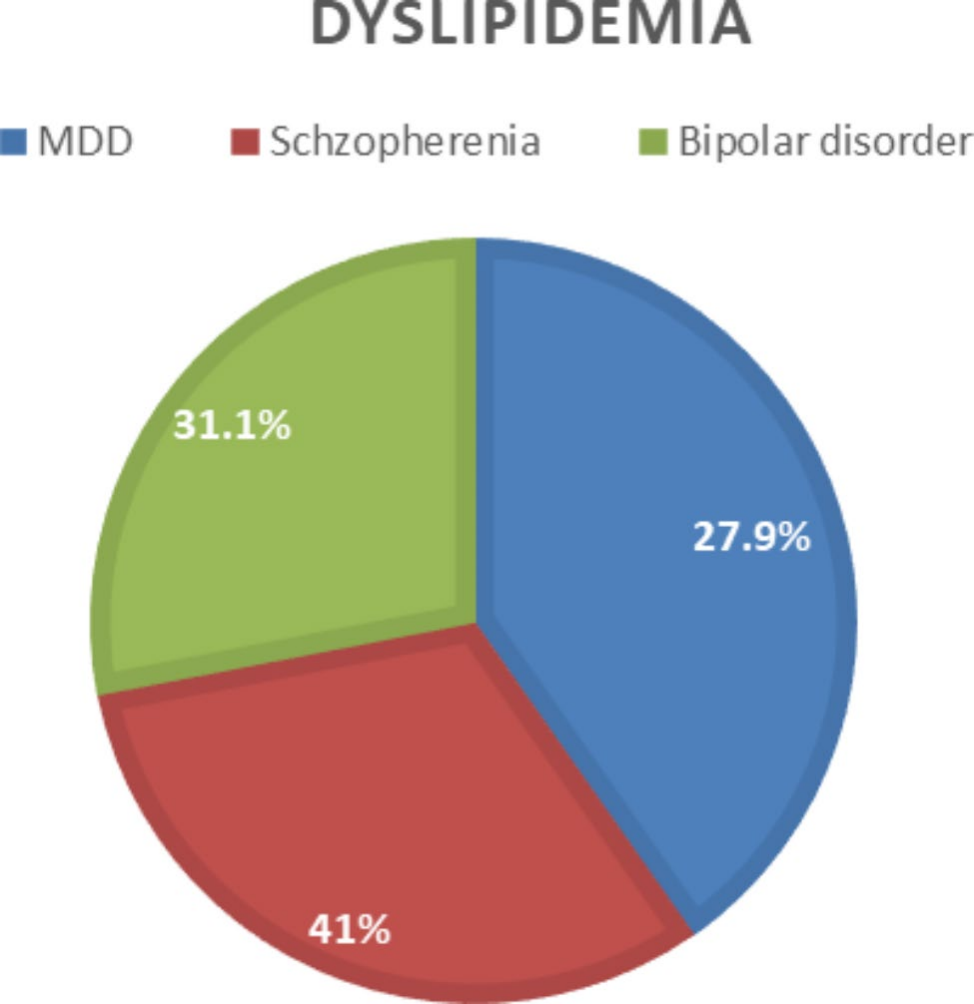
The magnitude of dyslipidemia among patients with severe mental illnesses,Dire Dawa,2021 (Note:MDD:Major Depressive Disorder)

The prevalence of dyslipidemia was highest in patients with schizophrenia (41%), followed by bipolar disorder (31.1%), and major depressive disorder (27.9%). There was a significant relationship between dyslipidemia and the patterns of serious mental illnesses (Fig. 1).

### Factors associated with dyslipidemia

All potential predictor variables were entered into a binary logistic regression to select candidate variables for multivariable regression. Variables with a p value of less than 0.25 in the bivariate analysis were included in the multivariable analysis. In multiple logistic regression, urban dwellers were six times (AOR = 6.14, 95% CI: 1.2, 16) more likely at risk of developing dyslipidemia compared to rural participants. Similarly, physically inactive participants were nearly two-times (AOR = 1.8, 95% CI: 1.1, 12.9) more likely to develop dyslipidemia compared to physically active study participants. Moreover, study participants who had raised body mass index were 2.1 times (AOR = 2.1, 95% CI: 1.17, 15.3) more likely having dyslipidemia than their counterparts (Table 3).

**Table 3:** Factors associated with dyslipidemia among seriously ill psychiatric patients, Dire Dawa, 2021

Variables	Category	Dyslipidemia		COR (95% CI)	AOR (95%CI)
		**Yes**	**No**		
Age (years)	<40	13(56.5%)	10 (43.5%)	ref	ref
	≥ 40	48(65.7%)	25(35.6%)	1.47(1.06–21.5)	0.47(.06,12.28)
Sex	Male	13(44.8%)	16(55.2%)	ref	ref
	Female	22(53.6%)	19(46.34%)	1.42(0.26,17.1)	.61 (0.11,27.6)
Education	Illiterate	23(51.1%)	22(48.9%)	2.7 (1.11,8.1)	1.3(0.1.0,9.5)
	Educated	38(74.5%)	13(25.49%)	ref	ref
Residency	rural	27(45.8%)	32(54.2%)	ref	ref
	urban	34(91.9%)	3(.9%)	13.43 (3.1,25)	6.14(1.2,16)*
Ever smoked	No	46(66.6%)	23(33.3%)	ref	ref
	yes	15(88.2%)	2(11.76%)	3.75(.79,16.6)	2.3(.36,7.8)
Ever taken alcohol	No	39(55.7%)	31(44.2%)	ref	ref
	yes	22(84.6%)	4(15.4%)	4.3 (0.6,11.10)	5.3(0.3,21.2)
Regular physical activity	yes	18(46.1%)	21(53.9%)	ref	ref
	No	43(75.4%)	14(24.56%)	3.58 (1.3,8.88)	3.8(1.1, 12.9)*
Duration of psychiatric illness (years)	< 10	22(53.6%)	19(46.4%)	ref	ref
	≥ 10	39(70.9%)	16(29.1%)	2.1(1.03,8.9)	1.8(0.20,8.18)*
BMI(kg/m2)	< 25	15(48.4%)	16(51.6%)	ref	ref
	≥ 25	46(70.8%)	19(29.2%)	2.58(1.91,10.3)	2.1(1.17,15.3)*
Blood pressure(SBP/DBP)	BP < 130/85	24(50%)	25(50%)	ref	ref
	BP ≥ 130/85	37(78.7%)	10(21.3%)	3.8(2.23,13.1)	0.78(.02,9.32)
Antipsychotics drug	First generation	15(45.5%)	18(54.5%)	ref	ref
	Second generation	46(73%)	17(27%)	3.25(1.33,9.45)	1.82(0.02,7.49)

SBP = systolic blood pressure, DBP = diastolic blood pressure, ref = reference, BMI = body mass index,*=p < 0.05, COR = crude odd ratio, AOR = adjusted odd ratio

## Discussion

Ethiopia is a developing nation, and like other sub-Saharan developing countries, it is going through a rapid epidemiological transition, i.e. under a double burden of communicable and non-communicable diseases [26]. Our study has two main findings. First, it confirms the very high prevalence rate of dyslipidemia in patients with severe mental illnesses. Second, urban residence, physical inactivity, and a raised body mass index were independently associated with dyslipidemia.

Furthermore, this study identified a higher prevalence of dyslipidemia among severely ill mental patients compared to non-exposed groups, which was 63.35% and 31.9%, respectively (p = 0.032). In addition to this, the components of lipid profile disorders such as hypercholesterolemia, raised LDL-c, and reduced HDL-c were significantly higher among mentally ill patients compared to non-exposed individuals. This might be cognitive deficit related lipid metabolic abnormalities brought on by the hypothalamic-adrenal axis disease, excessive substance use, and insufficient therapy that all contribute to mental patients developing dyslipidemia [27, 28]. It was comparable with other findings from southern Ethiopia (58.4%) [16], 68.1% in southwest Ethiopia [17], and (66.7%) northern Ethiopia [18]. On the other hand, this finding was relatively higher than studies conducted in China (19.66%) [12], Korea(12.6%) [29],and Saudi Arabia (8.5%) [30]. The disparities might be explained by sociodemographic, lifestyle, and patient care variability.

Likewise, urban residents were nearly six times more likely to have dyslipidemia compared to rural residents. This finding was consistent with the previous study reported in Central Ethiopia [31] and Saudi Arabia [13]. It is a known fact that most urban dwellers practice a sedentary lifestyle and an unhealthy diet, which in turn may lead to dyslipidemia. Similarly, studies participants who had a history of sedentary lifestyles were 3.8 times more likely to develop dyslipidemia compared to their counterparts. Similar findings were reported from Jimma Medical Center [17] and north eastern Ethiopia [19]. This might be due to the fact that physical activities contribute to lowered body lipid deposit status by utilizing as energy source, thus reducing the development of dyslipidemia and its complications. Furthermore, a higher body mass index (BMI ≥ 25 kg/m2) nearly quadrupled the likelihood of dyslipidemia compared to a normal body mass index. This finding is similar to studies conducted in Addis Ababa [32],Southwest Ethiopia [17],Northern Ethiopia [18] and Saudi [13]. This could be explained as raised body mass index causes insulin resistance which promotes increased flux of free fatty acids from the periphery to the liver stimulates excess hepatic lipid synthesis, which are responsible for the pathogenesis of dyslipidemia among these target groups [33].

### Limitations of the study

This study has some limitations. It included only severely ill mental patients, so the high dyslipidemia prevalence and its associated factors should not be generalized to the less serious psychiatric population. Due to a resource constraint, the dietary related information of the study participants was not assessed. Otherwise, the main strengths of the study are that the presence of a comparison group in this study made the demonstration of outcome measure risk more robust. This study was also supplemented by laboratory based biochemical analysis to enhance reliability.

## Conclusions

This study revealed that the prevalence of dyslipidemia is higher among mentally ill patients compared to non-mentally ill control study participants. Likewise, patients with severe mental illnesses had higher components of dyslipidemia such as hypercholesterolemia, higher low-density lipoprotein cholesterol, and reduced HDL-C levels compared to non-mentally ill control patients. Place of residence, physical inactivity, and raised BMI were significantly associated with dyslipidemia. Therefore, intensive screening of patients for dyslipidemia and its components is necessary during follow-up. Additionally, it is imperative that patients receive the proper intervention regarding lifestyle changes and the avoidance of risky behaviors.

## Data Availability

The manuscript contains all of the data that support the findings. The original data for this study is available from the corresponding author on a reasonable request.
